# Mapping where patients access primary care providers.

**DOI:** 10.23889/ijpds.v7i3.2068

**Published:** 2022-08-25

**Authors:** Eliot Frymire, Peter Gozdyra, Michael Green, Imaan Bayoumi, Richard Glazier, Liisa Jaakkimainen, Shahriar Khan, Tara Kiran, Kamila Premji

**Affiliations:** 1 Queens University; 2 ICES Toronto; 3 St. Michael’s Hospital; 4 Institute for Clinical Evaluative Sciences; 5 ICES Queens; 6 University of Ottawa

## Objectives

To gain an understanding of the attribution of patients to newly introduced Ontario Health Teams (OHT). OHTs are responsible for organizing and delivering health local care based on established connections between patients, their primary care providers, and hospitals. Furthermore, we aim to identify areas with poor geographic access to care.

## Approach

We used GIS analyses and maps to depict the attribution of patients to OHTs based on their uptake of primary care and hospital referral patterns. Residents of a specific local area can be attributed to different OHTs based on their prevailing health seeking choices. This leads to a creation of non-unique OHT ‘capture zones’, which may pose challenges in primary health care planning and delivery.

The range of spatial analyses and maps used in this study helps to overcome some of these limitations and provides healthcare administrators with important geographic layer of information not available through other data summary methods.

## Results

We used GIS analyses and maps to depict the attribution of patients to OHTs based on their uptake of primary care and hospital referral patterns. Residents of a specific local area can be attributed to different OHTs based on their prevailing health seeking choices. This leads to a creation of non-unique OHT ‘capture zones’, which may pose challenges in primary health care planning and delivery.

The range of spatial analyses and maps used in this study helps to overcome some of these limitations and provides healthcare administrators with important geographic layer of information not available through other data summary methods.

## Conclusion/Implications

These attribution maps and data tables are an essential resource for planners and decisions makers in identifying priorities within the regional provision of primary care. This knowledge is essential to a better understanding of health care needs of local populations, and to implementing improvements in health care access.

